# The ABC‐Stroke Risk Score and Effects of Atrial Fibrillation Screening on Stroke Prevention: Results From the Randomized LOOP Study

**DOI:** 10.1161/JAHA.123.032744

**Published:** 2024-02-14

**Authors:** Lucas Yixi Xing, Søren Zöga Diederichsen, Søren Højberg, Derk W. Krieger, Claus Graff, Ruth Frikke‐Schmidt, Pyotr G. Platonov, Morten S. Olesen, Axel Brandes, Lars Køber, Ketil Jørgen Haugan, Jesper Hastrup Svendsen

**Affiliations:** ^1^ Department of Cardiology Copenhagen University Hospital–Rigshospitalet Copenhagen Denmark; ^2^ Department of Cardiology Zealand University Hospital–Roskilde Roskilde Denmark; ^3^ Department of Cardiology Copenhagen University Hospital–Bispebjerg Copenhagen Denmark; ^4^ Department of Neurology, Mediclinic City Hospital Dubai United Arabic Emirates; ^5^ Department of Neuroscience Mohammed Bin Rashid University of Medicine and Health Science Dubai United Arabic Emirates; ^6^ Department of Health Science and Technology Aalborg University Gistrup Denmark; ^7^ Department of Clinical Biochemistry Copenhagen University Hospital–Rigshospitalet Copenhagen Denmark; ^8^ Department of Clinical Medicine, Faculty of Health and Medical Sciences University of Copenhagen Copenhagen Denmark; ^9^ Section II–Cardiology, Department of Clinical Sciences Lund University Lund Sweden; ^10^ Department of Biomedical Sciences, Faculty of Health and Medical Sciences University of Copenhagen Denmark; ^11^ Department of Clinical Research, Faculty of Health Sciences University of Southern Denmark Odense Denmark; ^12^ Department of Cardiology Odense University Hospital Odense Denmark; ^13^ Department of Cardiology Esbjerg Hospital–University Hospital of Southern Denmark Esbjerg Denmark

**Keywords:** atrial fibrillation, cardiac biomarker, risk score, screening, stroke, Atrial Fibrillation, Arrhythmias, Biomarkers, Ischemic Stroke

## Abstract

**Background:**

The ABC‐stroke score is a risk scheme for prediction of stroke or systemic embolism (SE) in atrial fibrillation (AF). This study sought to examine whether the score could be useful in predicting stroke in AF‐naïve individuals and risk stratifying for AF screening.

**Methods and Results:**

The LOOP (Atrial Fibrillation Detected by Continuous ECG Monitoring Using Implantable Loop Recorder to Prevent Stroke in High‐Risk Individuals) study randomized 6004 AF‐naïve individuals aged 70 to 90 years with stroke risk factors to either screening with an implantable loop recorder and anticoagulation upon detection of new‐onset AF episodes ≥6 minutes, or usual care. A total of 5781 participants had available ABC‐stroke score at baseline and were included in this secondary analysis: 4170 (72.1%) with an estimated stroke/SE risk ≤1%/year versus 1611 (27.9%) with an estimated stroke/SE risk >1%/year. Having an annual ABC‐stroke risk >1% was associated with stroke/SE, stroke/SE/cardiovascular death, and all‐cause death (hazard ratio, 1.82 [95% CI, 1.44–2.21], 2.17 [95% CI, 1.80–2.62], and 2.19 [95% CI, 1.87–2.56], respectively). For screening with implantable loop recorder versus usual care, no significant reduction in these study outcomes was obtained in any ABC‐stroke risk groups (*P*>0.0500 for all), with no signal toward interaction (*P*_interaction_>0.2500 for all). Similar findings were yielded when assessing the ABC‐stroke score as a continuous variable.

**Conclusions:**

In an elderly, AF‐naïve population with additional stroke risk factors, a higher ABC‐stroke score could identify individuals with increased stroke risk. However, this risk score may not be useful in pinpointing those more likely to benefit from AF screening and subsequent preventive treatment. These findings should be considered as hypothesis generating and warrant further study.

**Registration:**

URL: https://www.clinicaltrials.gov; unique identifier: NCT02036450.

**Nonstandard Abbreviations and Acronyms**
ESUS: embolic stroke of undetermined source
ILR: implantable loop recorder
SE: systemic embolism
TOAST: Trial of Org 10172 in Acute Stroke Treatment

**Clinical Perspective**

**What Is New?**

- In an elderly, high‐risk population without atrial fibrillation (AF), the ABC‐stroke risk score was associated with incident AF and stroke, with a better discriminative performance for stroke prediction than the conventional CHA_2_DS_2_‐VASc score.
- The effects of continuous AF screening versus usual care on stroke prevention were neutral regardless of the ABC‐stroke score, despite a positive correlation between the score and the risk of cardioembolic stroke specifically.

**What Are the Clinical Implications?**

- The ABC‐stroke score would not be useful in risk stratifying individuals for AF screening but might hold potential for guiding clinical decision making on anticoagulation initiation in patients with subclinical AF.

Atrial fibrillation (AF) is a well‐established risk factor for stroke,^1^, ^2^ but clear guidelines on screening for AF are currently lacking.^3^, ^4^ The LOOP Study (Atrial Fibrillation Detected by Continuous ECG Monitoring Using Implantable Loop Recorder to Prevent Stroke in High‐Risk Individuals) did not find a significant stroke risk reduction when elderly, high‐risk individuals were screened for subclinical AF using an implantable loop recorder (ILR), although more than a third of the screened participants were diagnosed with AF and initiated oral anticoagulation treatment.^5^ The CHA_2_DS_2_‐VASc score also did not appear to significantly modify the ILR screening effects on stroke prevention in the subgroup analysis. Thus, better tools for risk stratification are warranted to help identify individuals with a more clinically relevant AF phenotype likely to benefit from diagnosis and treatment.

Previous research suggests that cardiac biomarkers, such as high‐sensitivity cardiac troponins, are related to thromboembolic events and death in patients with AF.^5^, ^6^, ^7^, ^8^ Several prior studies have further demonstrated the superiority of the ABC‐stroke risk score over the CHA_2_DS_2_‐VASc score for stroke prediction in patient populations with AF.^9^, ^10^, ^11^, ^12^, ^13^ Therefore, the score may also have the potential to help identify the individuals more likely to benefit from AF screening and treatment. In this secondary analysis of the LOOP Study, we sought to examine the ABC‐stroke score for stroke prediction in a high‐risk, AF‐naïve population and for its association with the preventive effects of AF screening. Additional analyses were also carried out for cardiac troponins to provide further insights into the ABC‐stroke score.

## Methods

The data underlying this article cannot be shared publicly for ethical reasons, but the methodology will be shared on reasonable request to the corresponding author (J.H.S.) or the first author (L.Y.X.).

### LOOP Study

The LOOP Study (ClinicalTrials.gov identifier: NCT02036450) was a randomized controlled trial evaluating the effects of continuous AF screening on stroke prevention.^14^ The study design has been reported in detail previously.^15^ In brief, 6004 individuals aged 70 to 90 years, without known AF, and with ≥1 additional stroke risk factors (arterial hypertension, diabetes mellitus, heart failure, or prior stroke) were enrolled at 4 centers in Denmark and randomized 1:3 to ILR screening or usual care. In the ILR group, participants were offered oral anticoagulation therapy upon detection of any new‐onset AF episodes lasting ≥6 minutes confirmed by ≥2 senior cardiologists.

The LOOP Study was approved by the Regional Scientific Ethics Committee for the Capital Region of Denmark (H‐4‐2013‐025) and the Danish Data Protection Agency. The study was conducted in accordance with the Declaration of Helsinki, and all study participants provided oral and written informed consents at enrollment.

### Cardiac Biomarkers and ABC‐Stroke Risk Score

In the LOOP Study, blood samples were collected from the study participants at inclusion. High‐sensitivity troponin measurements were performed at the local laboratories of the enrolling centers, whereas the blood samples were transferred to 2 central hospital laboratories to measure NT‐proBNP (N‐terminal pro‐B‐type natriuretic peptide) levels. Due to the local difference in clinical routine troponin assays, 2 different types of cardiac troponin measurement were available for the study participants: either high‐sensitivity cardiac troponin T (TnT) or troponin I (TnI).

The ABC‐stroke risk score is a risk assessment scheme for prediction of stroke or systemic embolism (SE) in AF incorporating age, biomarkers (NT‐proBNP and high‐sensitivity TnT or TnI), and clinical history (prior stroke or transient ischemic attack).^9^ In the present study, to evaluate the baseline ABC‐stroke risk profile, we determined the annual ABC‐stroke risk in participants with available NT‐proBNP and cardiac troponin measurements at randomization, by using the risk regression equations derived from Hijazi et al.^9^ The ABC‐stroke risk groups were defined as low (≤1%) and medium‐high (>1%) 1‐year estimated risk of stroke/SE, which also aligns with the recommended risk threshold for initiating anticoagulation in patients with AF. As supplementary analyses, participants with available TnT or TnI measurements were also divided into subgroups on the basis of the respective median troponin concentrations.

### Study End Points and Assessment

As in the primary reporting of the LOOP Study, the primary outcome of this secondary analysis was stroke/SE, while secondary outcomes included (1) a composite end point of stroke, SE, or cardiovascular death, and (2) all‐cause death. A clinical end point committee blinded to randomization assignment was responsible for adjudication of the primary and secondary outcomes in accordance with predefined criteria.^15^ The stroke outcome did not include transient ischemic attack events and was further classified as ischemic versus nonischemic etiology and according to the TOAST (Trial of Org 10 172 in Acute Stroke Treatment) for ischemic stroke subtypes.^16^ When reporting, the ischemic strokes were divided into potentially cardioembolic strokes (defined as cardioembolic stroke or embolic stroke of undetermined source (ESUS)) and noncardioembolic strokes (defined as ischemic stroke due to large‐artery atherosclerosis, small‐vessel occlusion, or other determined etiologies). Additionally, AF diagnosis was also another outcome of interest in the present study, as the prognostic performance of the ABC‐stroke score might not necessarily translate into AF detection performance.

### Statistical Analysis

For baseline characteristics, categorical variables were summarized as frequency with percentage and continuous variables as mean with SD or median with interquartile range (IQR) where appropriate. Groupwise comparisons of the distributions were done by using χ^2^ test for categorical variables and Student's *t* test or Wilcoxon rank‐sum test for continuous variables.

Time‐to‐first‐event analyses were conducted for all study outcomes. Crude event rates (number of events per 100 person‐years) were calculated with the Poisson distribution, whereas cumulative incidences were determined using the Kaplan–Meier estimator or the Aalen‐Johansen estimator with death as a competing event.

For comparisons between ABC‐stroke risk groups and between troponin subgroups, the relative risks of study outcomes were examined in a multivariable cause‐specific Cox regression model, adjusted for sex, body mass index, weekly alcohol consumption, smoking burden (pack‐years), hypertension, diabetes, heart failure, ischemic heart disease, valvular heart disease, peripheral artery disease, age, and prior stroke (*the last 2 only for troponin subgroups*). For primary and secondary outcomes, the estimated ABC‐stroke risk and the high‐sensitivity cardiac troponin concentrations were further analyzed as continuous variables using restricted cubic spline regression in the multivariable Cox model, with knots located at the 5th, 35th, 65th, and 95th percentiles. Besides assessment of TnT and TnI separately, a pooled analysis was performed by first assigning each participant a percentile rank value of the respective troponin concentrations and thereafter evaluating the risks of primary and secondary outcomes according to the overall troponin percentile rank as a continuous variable. Moreover, to compare the predictive performance of the ABC‐stroke score with the CHA_2_DS_2_‐VASc score, a time‐dependent receiver operating characteristic (ROC) curve analysis was conducted for 6‐year outcome of stroke/SE, and the area under the curve (AUC) was calculated for these 2 risk scores.^17^ Also, the ROC curve analysis was repeated for each component of the ABC‐stroke score (ie, age, biomarkers, and prior history of stroke or transient ischemic attack), where the AUC of the biomarker component was determined using a model with NT‐proBNP and the overall troponin percentile rank.

Finally, the relationships between ILR screening effects on study outcomes and ABC‐stroke risk groups or troponin subgroups were examined by using cause‐specific Cox regression models, wherein an interaction term was incorporated to further test the potential effect modification between randomization assignment and subgroups. The ABC‐stroke risk and the troponin concentrations were also assessed as continuous variables for their associations with the effects of ILR screening versus usual care on primary and secondary outcomes. The continuous troponins were evaluated both as TnT and TnI separately and as the overall troponin percentile rank in a pooled analysis as described above.

The Cox proportional‐hazards assumption was checked using scaled Schoenfeld residuals and any violations led to stratification of the relevant variables to allow different baseline hazards. The data analysis was performed using R version 4.2.2 (R Core Team). A 2‐sided *P* ≤0.05 was applied to define the statistical significance.

## Results

Of 6004 study participants enrolled in the LOOP Study, 5948 (99.1%) had available high‐sensitivity troponin measurement at baseline: 3313 with TnT and 2635 with TnI. The median concentration of high‐sensitivity troponin was 13 ng/L for TnT and 15 ng/L for TnI. Due to missing NT‐proBNP (n=167), the ABC‐stroke score was calculated in 5781 (97.2%) of 5948. Table 1 summarizes the baseline characteristics according to ABC‐stroke risk groups. Participants with an estimated ABC‐stroke risk >1%/year (the medium‐high‐risk group) at baseline tended to be older and more likely men and had higher smoking burden than those in the low‐risk group. In terms of comorbidities, established cardiovascular diseases, such as heart failure, previous stroke, ischemic heart disease, valvular heart disease, and peripheral artery disease, were also more prevalent in participants with higher ABC‐stroke score. Further, compared with those at low predicted risk, the medium‐high ABC‐stroke risk group had a higher NT‐proBNP concentration (median 25.0 [IQR, 12.0–61.9] versus 13.7 [IQR, 8.0–22.4] pmol/L) and were more likely to have a baseline high‐sensitivity troponin measurement above median (54.6% versus 39.2%).

**Table 1:** Baseline Characteristics According to ABC‐Stroke Risk Groups

	Total (n=5781)	Baseline ABC‐stroke risk		
		Risk ≤1%/year (n=4170)	Risk >1%/year (n=1611)	*P* value
ILR assignment, n (%)	1453 (25.1)	1047 (25.1)	406 (25.2)	0.9682
Male sex, n (%)	3033 (52.5)	2055 (49.3)	978 (60.7)	<0.0001
Age, y, mean (SD)	74.8 (4.1)	74.4 (3.8)	75.8 (4.7)	<0.0001
Alcohol consumption, standard units per week, median (IQR)	5 (1–10)	5 (1–10)	5 (1–10)	0.7852
Smoking pack years, median (IQR)	6 (0–28)	5 (0–26)	10 (0–31)	<0.0001
Systolic blood pressure, mmHg, mean (SD)	149.9 (19.5)	149.8 (19.1)	150.3 (20.5)	0.4030
Body mass index, kg/m^2^, mean (SD)	27.6 (4.6)	27.9 (4.7)	27.0 (4.3)	<0.0001
CHA_2_DS_2_‐VASc score, mean (SD)	3.8 (1.2)	3.4 (1)	4.8 (1.1)	<0.0001
Comorbidities, n (%)				
Arterial hypertension	5239 (90.6)	3894 (93.4)	1345 (83.5)	<0.0001
Diabetes mellitus	1644 (28.4)	1274 (30.6)	370 (23.0)	<0.0001
Heart failure	262 (4.5)	146 (3.5)	116 (7.2)	<0.0001
Previous stroke	1016 (17.6)	34 (0.8)	982 (61.0)	<0.0001
Chronic ischemic heart disease	774 (13.4)	474 (11.4)	300 (18.6)	<0.0001
Valvular heart disease	235 (4.1)	149 (3.6)	86 (5.3)	0.0030
Peripheral artery disease	156 (2.7)	92 (2.2)	64 (4.0)	0.0003
Concomitant medications, n (%)				
β Blockers	1493 (25.8)	1030 (24.7)	463 (28.7)	0.0019
Calcium channel blockers	2161 (37.4)	1636 (39.2)	525 (32.6)	<0.0001
Renin–angiotensin inhibitors	3832 (66.3)	2864 (68.7)	968 (60.1)	<0.0001
Diuretics	1938 (33.5)	1416 (34.0)	522 (32.4)	0.2750
Platelet inhibitors	2813 (48.7)	1539 (36.9)	1274 (79.1)	<0.0001
Statins	3349 (57.9)	2219 (53.2)	1130 (70.1)	<0.0001
Insulins	463 (8.0)	369 (8.8)	94 (5.8)	0.0002
Other antidiabetic drugs	1233 (21.3)	962 (23.1)	271 (16.8)	<0.0001
Digitalis	8 (0.1)	4 (0.1)	4 (0.2)	0.3160

Values are presented as n (%), mean (SD), or median (IQR). Missing observations: alcohol consumption, n=3; systolic blood pressure, n=6. ILR indicates implantable loop recorder; and IQR, interquartile range.

### Primary and Secondary Outcomes

**Figure 1:**
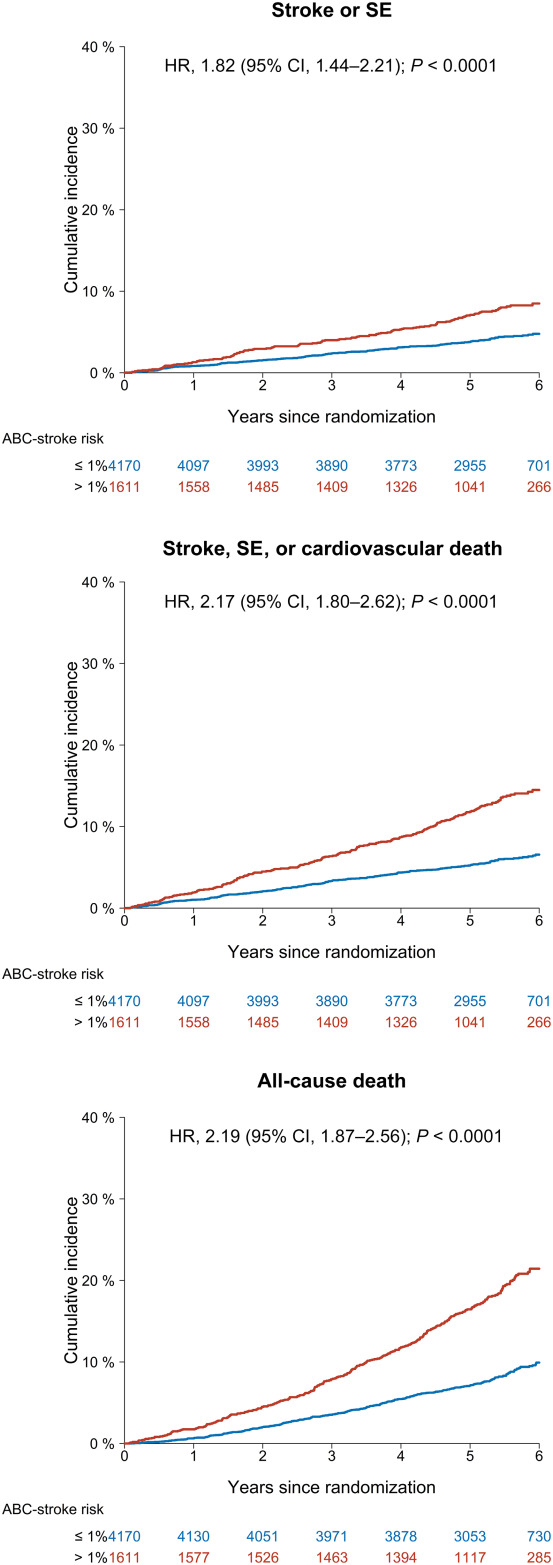
Cumulative incidences of primary and secondary outcomes according to the annual ABC‐stroke risk. Absolute risks of stroke/SE, stroke/SE/cardiovascular death, and all‐cause death in the entire study cohort according to the ABC‐stroke risk groups. Cumulative incidences were plotted using the Kaplan–Meier estimator for all‐cause death and the Aalen–Johansen estimator for other outcomes with death as a competing event. HRs and *P* values were determined in cause‐specific Cox models adjusted for sex, body mass index, weekly alcohol consumption, smoking pack‐years, hypertension, diabetes, heart failure, valvular heart disease, ischemic heart disease, and peripheral artery disease. HR indicates hazard ratio; and SE, systemic embolism.

**Figure 2:**
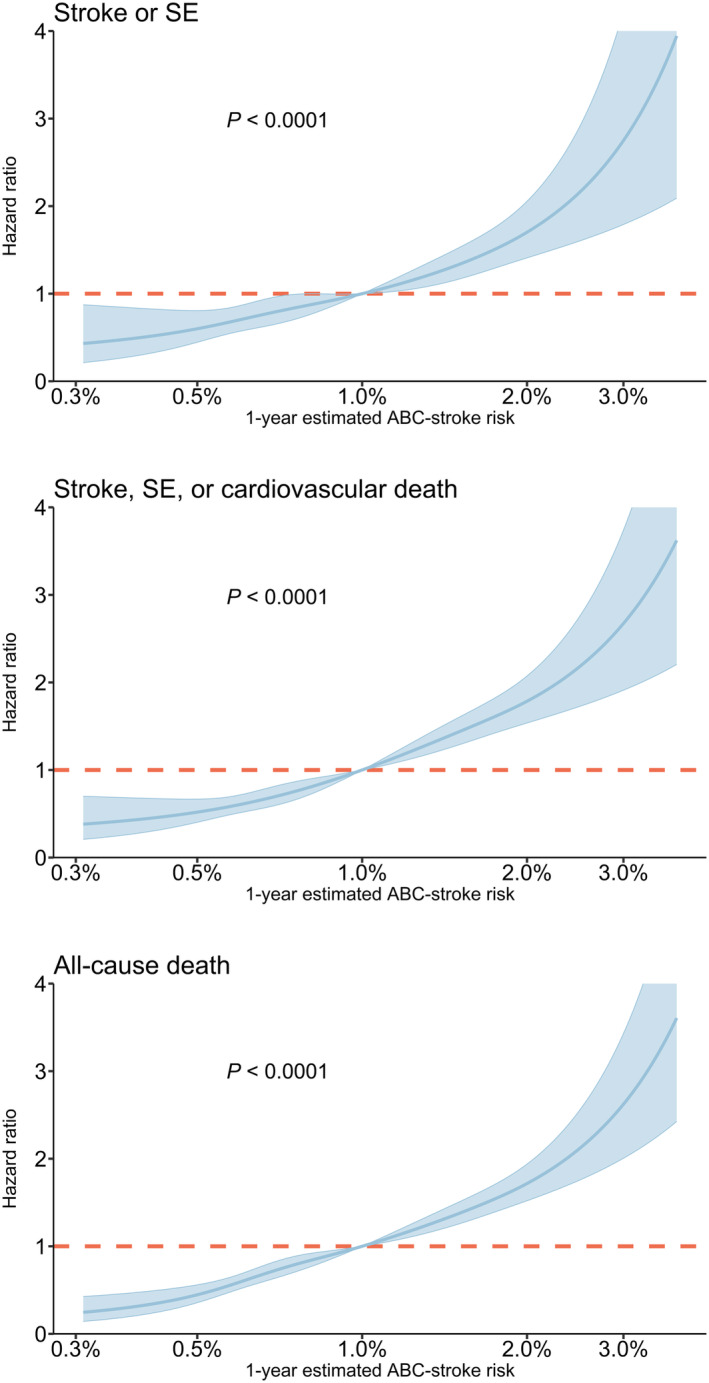
The associations of primary and secondary outcomes with the estimated ABC‐stroke risk. Risks of stroke/SE, stroke/SE/cardiovascular death, and all‐cause death in the entire study cohort according to the estimated ABC‐stroke risk at baseline. Hazard ratios were determined with the estimated risk of 1% by ABC‐stroke score as reference, in cause‐specific Cox models adjusted for sex, body mass index, weekly alcohol consumption, smoking pack‐years, hypertension, diabetes, heart failure, valvular heart disease, ischemic heart disease, and peripheral artery disease. The colored areas represent 95% CIs. SE indicates systemic embolism.

**Figure 3:**
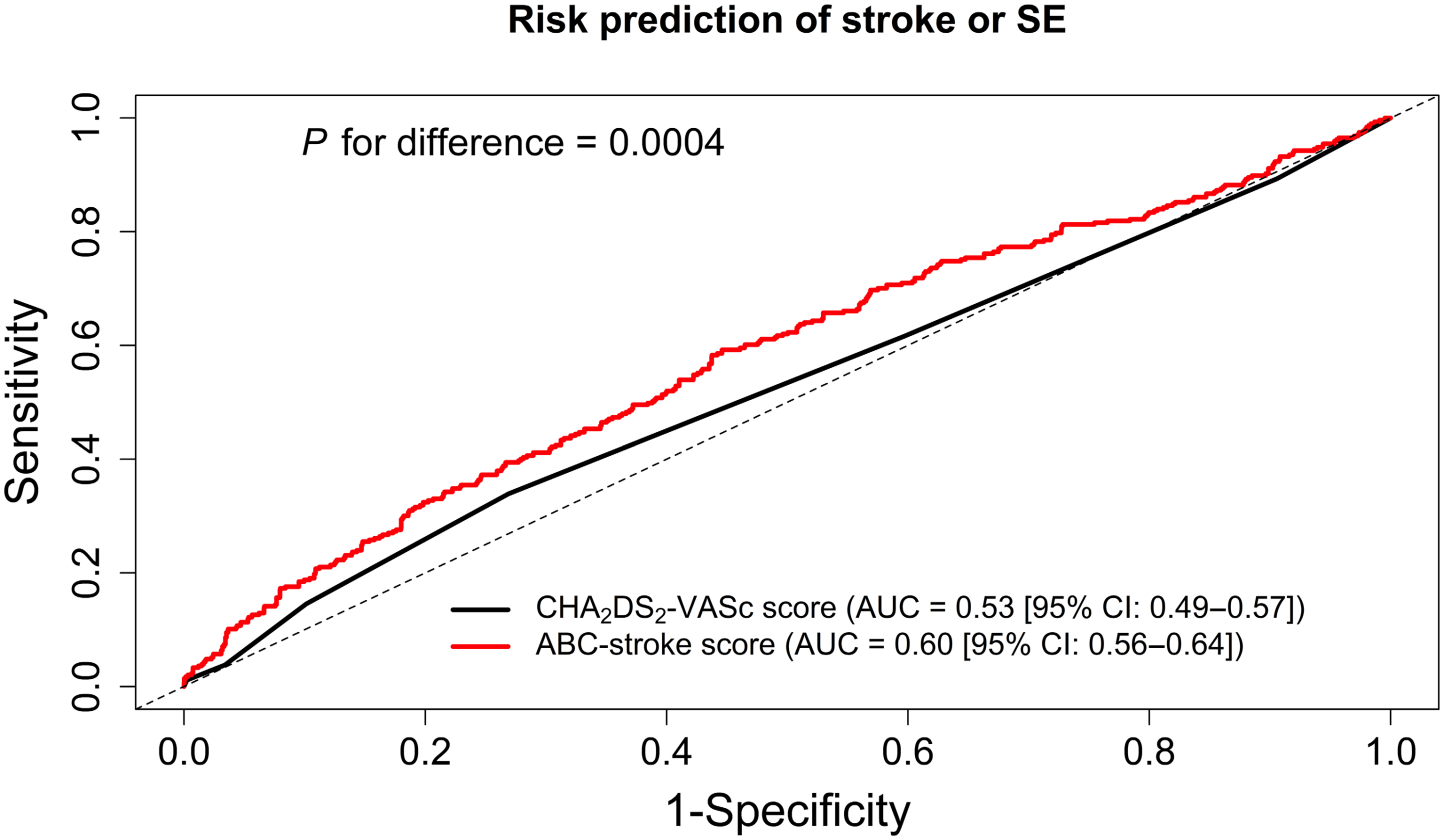
Time‐dependent ROC curve analysis for risk prediction of the primary outcome. Time‐dependent ROC curves for risk prediction of stroke or SE using the baseline CHA_2_DS_2_‐VASc score and ABC‐stroke risk score. The analysis was conducted for 6‐year outcome of stroke or SE in the study population with an available ABC‐stroke score at baseline. AUC indicates area under the curve; ROC, receiver operating characteristic; and SE, systemic embolism.

Among the 5781 participants with available ABC‐stroke score at baseline, 307 (5.3%) had stroke/SE (304 strokes and 3 SEs) during a median follow‐up time of 5.4 years [IQR, 4.9–5.8]: 181 in the low ABC‐stroke risk group and 126 in the medium‐high‐risk group. Figure 1 shows the cumulative incidences of the primary and secondary outcomes according to the ABC‐stroke risk groups. The 1‐year cumulative incidence of stroke/SE was 0.84% (95% CI, 0.56–1.12) for the low ABC‐stroke risk group (≤1%/year) and 1.31% (95% CI, 0.75–1.86) for the medium‐high‐risk group (>1%/year). The crude event rates of stroke/SE were 0.84 (95% CI, 0.72–0.97) and 1.60 (95% CI, 1.33–1.90) per 100 person‐years among participants with low versus medium‐high ABC‐stroke risk, respectively. This corresponded to a hazard ratio (HR) of 1.82 (95% CI, 1.44–2.21). For secondary outcomes, both the risks of stroke/SE/cardiovascular death and all‐cause death were significantly increased among participants in the medium‐high ABC‐stroke risk group (HR, 2.17 [95% CI, 1.80–2.62] and 2.19 [95% CI, 1.87–2.56], respectively). When further assessed as a continuous variable, the estimated ABC‐stroke risk showed a positive correlation with both primary and secondary outcomes (Figure 2). The time‐dependent ROC analysis also revealed a better predictive value of the estimated ABC‐stroke risk at baseline for stroke/SE than the CHA_2_DS_2_‐VASc score, as illustrated in Figure 3. The AUC was 0.60 (95% CI, 0.56–0.64) for the ABC‐stroke risk score versus 0.53 (95% CI, 0.49–0.57) for the CHA_2_DS_2_‐VASc score; *P*=0.0004. The AUCs for each component of the ABC‐stroke score are presented in Table S1. The discriminative performance of the ABC‐stroke score was mainly upheld by the biomarkers and the presence of prior stroke or transient ischemic attack. Also, age and prior stroke history did not appear to significantly improve the stroke prediction when added to the biomarker component (*P*=0.3032).

In terms of stroke etiology, 264 (4.6%) of 5781 participants had ischemic stroke: 105 (6.5%) in the medium‐high ABC‐stroke risk group versus 159 (3.8%) in the low‐risk group (HR, 1.68 [95% CI, 1.30–2.18]). Further for stroke subtypes according to the TOAST classification, 113 (2.0%) had cardioembolic stroke or ESUS, and 151 (2.6%) had noncardioembolic ischemic stroke. When compared with the low ABC‐stroke risk group, participants in the medium‐high‐risk group were at significantly increased risk of cardioembolic stroke or ESUS (HR, 2.24 [95% CI, 1.52–3.31]) but not of noncardioembolic stroke (HR, 1.35 [95% CI, 0.95–1.91]).

Figures S1 and S2 display cumulative incidence curves of primary and secondary outcomes according to troponin subgroups among participants with available TnT measurement (n=3313) and participants with available TnI measurement (n=2635), respectively. The presence of TnI >15 ng/L at baseline was related to a significantly increased risk of stroke/SE compared with lower concentrations (HR, 1.84 [95% CI, 1.08–3.15]), whereas participants with TnT >13 ng/L were at numerically higher risk of the primary outcome than those with lower TnT (HR, 1.21 [95% CI, 0.88–1.67]). With respect to secondary outcomes, both TnT above median and TnI above median were associated with significantly higher risks of stroke/SE/cardiovascular death and all‐cause death. The crude event rates and the relative risks of primary and secondary outcomes according to troponin subgroups are provided in Table 2. Additionally, the restricted cubic spline regression analysis also demonstrated significant associations of primary and secondary outcomes with continuous TnT (Figure S3) and with continuous Tni ( *P* ≤0.0500 for all; Figure S4). Similar relationship patterns were observed when the troponin measurements were evaluated as the overall percentile rank in a pooled analysis (Figure S5).

**Table 2:** Primary and Secondary Outcomes According to ABC‐Stroke Risk Groups and Troponin Subgroups

	Variables		Crude event rate (95% CI)	Hazard ratio (95% CI)	*P* value
Stroke or SE	1‐Year ABC‐stroke risk	≤1%	0.84 (0.72–0.97)	1.82 (1.44–2.21)	<0.0001
		>1%	1.60 (1.33–1.90)		
	High‐sensitivity TnT	≤13 ng/L	1.01 (0.84–1.20)	1.21 (0.88–1.67)	0.2468
		>13 ng/L	1.49 (1.16–1.90)		
	High‐sensitivity TnI	≤15 ng/L	0.84 (0.69–1.02)	1.84 (1.08–3.15)	0.0252
		>15 ng/L	1.64 (0.94–2.67)		
Stroke, SE, or cardiovascular death	1‐Year ABC‐stroke risk	≤1%	1.16 (1.03–1.32)	2.17 (1.80–2.62)	<0.0001
		>1%	2.74 (2.38–3.13)		
	High‐sensitivity TnT	≤13 ng/L	1.35 (1.16–1.57)	1.62 (1.25–2.09)	0.0002
		>13 ng/L	2.65 (2.20–3.18)		
	High‐sensitivity TnI	≤15 ng/L	1.29 (1.09–1.50)	2.09 (1.39–3.13)	0.0004
		>15 ng/L	2.98 (2.00–4.28)		
All‐cause death	1‐Year ABC‐stroke risk	≤1%	1.62 (1.45–1.79)	2.19 (1.87–2.56)	<0.0001
		>1%	3.83 (3.42–4.28)		
	High‐sensitivity TnT	≤13 ng/L	1.60 (1.39–1.84)	1.94 (1.57–2.41)	<0.0001
		>13 ng/L	3.97 (3.41–4.59)		
	High‐sensitivity TnI	≤15 ng/L	1.98 (1.74–2.25)	1.55 (1.09–2.21)	0.0159
		>15 ng/L	3.56 (2.50–4.93)		

Crude event rates are presented as event number per 100 person‐years. Hazard ratios were estimated in cause‐specific Cox regression models adjusted for sex, body mass index, weekly alcohol consumption, smoking pack‐years, hypertension, diabetes, heart failure, valvular heart disease, ischemic heart disease, peripheral artery disease, age, and prior stroke (*the last 2 only for troponin subgroups*). SE indicates systemic embolism; TnI, troponin I; and TnT, troponin T.

### ILR Screening Effects on Primary and Secondary Outcomes

**Figure 4:**
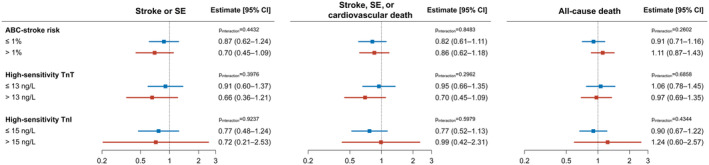
ILR screening effects on primary and secondary outcomes according to the annual ABC‐stroke risk and troponin concentrations at baseline. Forest plot of hazard ratios of stroke/SE, stroke/SE/cardiovascular death, and all‐cause death for ILR screening versus usual care according to ABC‐stroke risk groups, high‐sensitivity TnT subgroups, and high‐sensitivity TnI subgroups. Hazard ratios and *P* values for interaction were determined in cause‐specific Cox models. ILR indicates implantable loop recorder; SE, systemic embolism; TnI, troponin I; and TnT, troponin T.

Figure 4 depicts the effects of ILR screening versus usual care on primary and secondary outcomes according to ABC‐stroke risk groups and troponin subgroups. No significant effect modifications between ILR screening and ABC‐stroke risk groups or troponin subgroups were found (*P*_interaction_>0.2500 for all). The screening effects on stroke/SE, stroke/SE/cardiovascular death, and all‐cause death also remained neutral regardless of ABC‐stroke risk groups and troponin subgroups, respectively. Further with the ABC‐stroke score and the troponin concentrations analyzed as continuous variables, similar effect patterns on primary and secondary outcomes were present, with no signal toward interaction. Figures S6 and S7 portray ILR screening effects according to the continuous ABC‐stroke risk and troponin percentile rank, respectively. Similar observations were made when assessing the screening effects on ischemic stroke, cardioembolic stroke or ESUS, and noncardioembolic stroke, separately, across the ABC‐stroke risk groups, with no significant interaction detected (*P*_interaction_>0.5000 for all; see Table S2).

### AF Diagnosis

**Figure 5:**
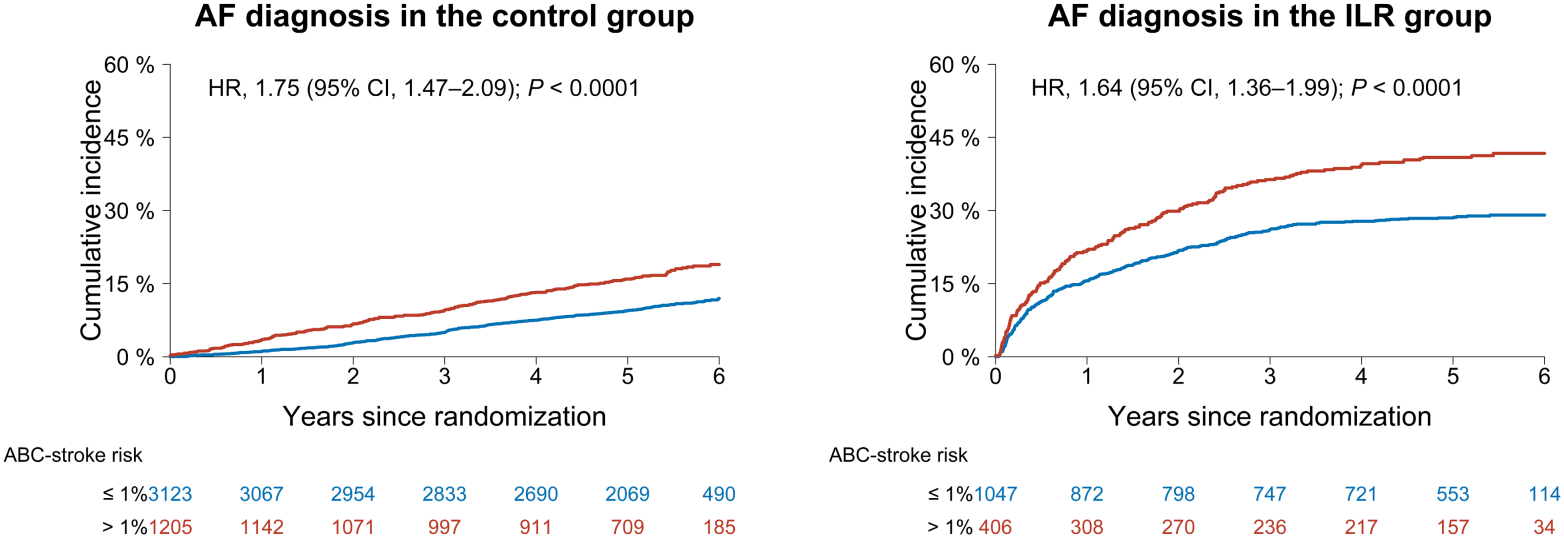
Cumulative incidences of AF diagnosis according to the annual ABC‐stroke risk at baseline. Absolute risk of AF diagnosis in the control group and in the ILR group, according to the ABC‐stroke risk groups. Cumulative incidences were plotted using the Aalen–Johansen estimator with death as a competing event. HRs and *P* values were determined in cause‐specific Cox models adjusted for sex, body mass index, weekly alcohol consumption, smoking pack‐years, hypertension, diabetes, heart failure, valvular heart disease, ischemic heart disease, and peripheral artery disease. AF indicates atrial fibrillation; HR, hazard ratio; and ILR, implantable loop recorder.

AF was diagnosed in 1007 (17.4%) of 5781 participants with available ABC‐stroke score: 540 (12.5%) of 4328 in the control group versus 467 (32.1%) of 1453 in the ILR group. The presence of an estimated ABC‐stroke risk >1%/year at baseline was associated with higher incidence of AF diagnosis in either randomization groups (HR, 1.75 [95% CI, 1.47–2.09] in the control group and 1.64 [95% CI, 1.36–1.99] in the ILR group; Figure 5). When compared with usual care, a significant increase in AF diagnosis was obtained by ILR screening both in the low ABC‐stroke risk group (HR, 3.26 [95% CI, 2.79–3.82]) and the medium‐high‐risk group (HR, 3.03 [95% CI, 2.47–3.71]).

Figures S8 and S9 show cumulative incidence curves of AF diagnosis according to troponin subgroups. Participants with TnT or TnI above median were more likely to be diagnosed with AF in either randomization group, compared with those having lower concentrations at baseline. For ILR screening versus usual care, the incidence of AF diagnosis was also increased both in participants with troponin measurements over median (HR, 3.05 [95% CI, 2.38–3.91] for TnT and 2.72 [95% CI, 1.66–4.45] for TnI) and in those with lower concentrations (HR, 4.29 [95% CI, 3.45–5.34] for TnT and 2.67 [95% CI, 2.18–3.28] for TnI).

## Discussion

In elderly, AF‐naïve individuals with additional stroke risk factors enrolled in a large, randomized trial for AF screening, we reported the following major findings: (1) Both the ABC‐stroke risk score and high‐sensitivity cardiac troponins were associated with incident AF, stroke, and death; (2) the discriminative performance of the ABC‐stroke score for stroke prediction was significantly better than the conventional CHA_2_DS_2_‐VASc score; and (3) the effects of ILR screening versus usual care on stroke prevention were neutral regardless of the ABC‐stroke score or the troponin concentrations at baseline.

The ABC‐stroke risk score is a risk stratification scheme encompassing age, biomarkers (NT‐proBNP and high‐sensitivity TnT or TnI), and clinical history (previous history of stroke or transient ischemic attack).^9^ The score has been validated in several clinical trial cohorts of patients with AF as well as in an observational study cohort of patients admitted with unstable AF.^9^, ^10^, ^11^, ^12^, ^13^ To the best of our knowledge, the present study is the first to evaluate the ABC‐stroke score in nonanticoagulated, AF‐naïve individuals. Our data not only show that the predicted risk by ABC‐stroke score fits reasonably well with the observed stroke risk, but also confirm a positive correlation between the risk score and incident stroke. Indeed, NT‐proBNP and cardiac troponins (the B component of the ABC‐stroke score) have already been linked to stroke in large community‐based studies,^18^, ^19^, ^20^, ^21^ besides the A and C components of age and stroke history being well‐documented risk factors and also being part of the CHA_2_DS_2_‐VASc score.^4^, ^22^ These previous findings are further corroborated by our observation of participants with higher cardiac troponin concentrations having higher stroke risk, which was also the case for higher NT‐proBNP as reported by a recent study.^23^ In addition, we found an increased AF risk among participants with higher ABC‐stroke score and with higher troponin concentrations, which could partly explain the elevated stroke risk in these participant groups. This notion is indeed supported by our finding of a higher ABC‐stroke score being associated with increased risk of cardioembolic stroke or ESUS, but not of noncardioembolic ischemic stroke. It is worth mentioning that the ABC‐stroke score also appeared to outperform the CHA_2_DS_2_‐VASc score for stroke risk prediction even in this study population without known AF at baseline (AUC 0.60 versus 0.53). Our results are comparable to those derived from large, randomized trials with AF patients.^9^, ^10^, ^11^, ^12^ In the original validation cohort of the STABILITY (Stabilization of Atherosclerotic Plaque by Initiation of Darapladib Therapy) trial, the discriminative ability for stroke prediction was 0.66 for the ABC‐stroke score and 0.58 for the CHA_2_DS_2_‐VASc score.^9^ Despite its excellent capability of identifying individuals with a truly low risk of stroke, the CHA_2_DS_2_‐VASc score is known to have only a modest discriminative performance when it comes to risk stratification among those at high risk.^4^ More importantly, even in our LOOP study population with a CHA_2_DS_2_‐VASc score ≥2, the ABC‐stroke score still successfully distinguished and predicted the low‐risk participants (≤1%/year). These findings are in great agreement with prior studies demonstrating an improved stroke prediction by adding NT‐proBNP and troponins to the CHA_2_DS_2_‐VASc score.^5^, ^6^ Thus, it could be postulated that cardiac biomarkers may reflect preclinical cardiovascular phenotypes and thereby provide additional prognostic information beyond clinical variables. Moreover, the CHA_2_DS_2_‐VASc score consists mainly of binary, irreversible entries of clinical history and does not take into account the degrees of the different disease components or the impact of treatment response, for stroke risk assessment. In this aspect, circulating cardiac biomarkers would speculatively serve as superior indicators of current disease states in patients, enabling a more precise risk estimation. The use of the ABC‐stroke score could therefore potentially contribute to the advancement of precision health care approaches for stroke prophylaxis in AF. However, an ongoing ABC‐AF (ABC‐Scores for Reduction of Stroke and Mortality in Atrial Fibrillation) study is currently evaluating the anticoagulation treatment strategy based on the ABC‐stroke score compared with the CHA_2_DS_2_‐VASc score in patients with AF and may help to clarify the clinical utility of this biomarker‐guided approach for stroke prevention.^4^

Current guidelines mandate the use of the CHA_2_DS_2_‐VASc score for the evaluation of stroke risk and the decision‐making process regarding anticoagulation initiation in patients with AF.^4^, ^24^ However, in the main reporting of the LOOP study, ILR screening effects on stroke prevention did not seem to change with the CHA_2_DS_2_‐VASc score.^14^ Similarly, we found no signal toward the ABC‐stroke score being able to discriminate between individuals with or without benefit from AF screening in the present study, albeit its associations with AF and with stroke. The screening effects remained neutral regardless of the ABC‐stroke score at baseline. Hence, the risk scheme does help to identify individuals at increased risks of AF and stroke, but it may not be useful in selecting for AF screening to prevent stroke. In fact, the screening may lead to various downstream interventions and actions, including anticoagulation treatment, which can have varying impact on the overall screening benefits. Therefore, a relevant risk profile should not be the sole factor considered to instigate screening. However, while the ABC‐stroke score may not be effective in risk stratification for AF screening, it might still hold potential for guiding clinical decision making on anticoagulation initiation for subclinical AF, as the score was specifically associated with cardioembolic stroke and ESUS according to our results. In terms of screening effects, several possible explanations for our negative results could exist. The observed slightly lower relative risk estimate for ILR versus control in the medium‐high ABC‐stroke risk group was more likely upheld by higher NT‐proBNP only, as a linkage between NT‐proBNP and effects of AF screening has been reported by a recent analysis of the LOOP study.^23^ For cardiac troponins, participants with higher concentrations did not seem to derive any benefit from ILR screening in the present study. As an established biomarker for acute myocardial infarction,^25^, ^26^ cardiac troponins could also very well represent the atherosclerotic risk profile in clinically stable individuals. A Swedish community‐based study of elderly subjects found the troponin elevation to be related to greater atherosclerotic burden in the carotid arteries as indicated by the number of arteries with plaques and the plaque size,^27^ while Zethelius et al reported a positive correlation between cardiac troponin and the risk of coronary artery disease in elderly, healthy men.^28^ This could partially account for the lacking response on AF screening among participants with elevated troponins, as the presence of competing risk factors for stroke might prevent them from benefitting from AF screening. The same also applies to participants with a higher predicted ABC‐stroke risk, who were more likely to have cardiovascular diseases at baseline, including prior stroke with a prevalence of 61.0% in the medium‐high‐risk group. Indeed, previous secondary analyses of the LOOP study demonstrated in patients with established cardiovascular disease or prior stroke having less benefit of screening, despite greater screening yields and higher stroke rates overall.^29^, ^30^ Besides, another possible explanation for the neutral screening effect in the medium‐high ABC‐stroke risk group could be that the existing patient care regimens in patients with a high burden of cardiovascular comorbidities might already be effective in detecting clinically relevant AF, and thus, ILR screening would not contribute to additional stroke prevention among them. This is arguably supported by our finding of a comparable, significant risk increase of AF diagnosis across ABC‐stroke risk groups in the control group as in the ILR group.

### Limitations

Several study limitations warrant considerations. First, this was a secondary analysis of a randomized trial and therefore, our results should be considered hypothesis generating only. Second, due to the limited number of participants within each subgroup, our study might be underpowered to detect smaller but still clinically relevant risk reductions. Third, our study cohort consisted of elderly individuals aged 70 to 90 years recruited from a population of primarily White individuals, which would limit the generalizability of our findings to other age and race groups. Fourth, as we only have TnT and TnI measurements for subsets of our participants, the study might be underpowered to detect relevant associations with cardiac troponins. However, to address this limitation, we had also conducted a pooled analysis for all troponin measurement by using the overall percentile rank as an attempt to maximize the statistical power.

## Conclusions

In elderly, AF‐naïve individuals with additional stroke risk factors, the ABC‐stroke risk score was associated with AF diagnosis, stroke, and death, further demonstrating a better discriminative performance for stroke prediction compared with the CHA_2_DS_2_‐VASc score. However, the ABC‐stroke score did not modify any preventive effects of AF screening versus usual care. This indicates that the ABC‐stroke score does indeed help to identify the population with increased risks of AF and stroke, but that these may not have any benefit from AF screening on stroke prevention. These findings should be considered as hypothesis generating and warrant further study.

## Sources of Funding

The LOOP Study was supported by the Innovation Fund Denmark (grant no. 12–1352259), The Research Foundation for the Capital Region of Denmark, the Danish Heart Foundation [grant no. 11‐04‐R83‐A3363‐22625], Aalborg University Talent Management Program, Arvid Nilssons Fond, Skibsreder Per Henriksen, R og Hustrus Fond, the European Union's Horizon 2020 program (grant no. 847770 to the AFFECT‐EU consortium), Læge Sophus Carl Emil Friis og hustru Olga Doris Friis' Legat, and an unrestricted grant from Medtronic. The employment of the first author, LYX, is funded by the European Union's Horizon 2020 program (grant no. 847770) through the AFFECT‐EU consortium.

## Disclosures

Dr Svendsen is a member of Medtronic advisory boards and has received speaker honoraria and research grants from Medtronic in relation to this work and outside this work. Dr Diederichsen is a part‐time employee of VitalBeats and advisor at Bristol‐Myers Squibb/Pfizer, not related to this work. Dr Krieger is a Medtronic Focus Group member. Dr Frikke‐Schmidt reports speaker honorarium and consultant fees from Novo Nordisk. Dr Brandes reports research grants from the Region of Zealand, the Canadian Institutes of Health Research, the Danish Heart Foundation, and Theravance, and speaker honoraria from Boehringer Ingelheim and Bristol‐Myers Squibb not related to this work. Dr Køber reports speaker honoraria from Novo Nordisk, AstraZeneca, Novartis, and Boehringer, not related to this work. The remaining authors have no disclosures to report.

## Supporting information

Tables S1–S2
Figures S1–S9
