# Supplementary material for: The ABC‐Stroke Risk Score and Effects of Atrial Fibrillation Screening on Stroke Prevention: Results From the Randomized LOOP Study
Source: J Am Heart Assoc. 2024 Feb 14;13(4):e032744. doi: 10.1161/JAHA.123.032744 (PMC11010080; doi:10.1161/JAHA.123.032744)
Supplement: Supplementary file 1 — Tables S1–S2 Figures S1–S9 [file JAH3-13-e032744-s001.pdf]

SUPPLEMENTAL MATERIAL

Table S1. **Time-dependent AUC for each component of the ABC-stroke score.**

	**Time-dependent AUC**	**p-value for difference compared with the ABC-stroke score**
**Age**	0.50 [0.45-0.54]	<0.0001
**Biomarkers**	0.58 [0.54-0.62]	0.3032
**Clinical history of stroke or transient ischemic stroke**	0.56 [0.53-0.59]	0.0106

The time-dependent ROC analysis was performed in the study participants with available ABC-stroke score at baseline. The AUC for the B-component was estimated by using a model with NT-proBNP and the percentile rank of high-sensitivity troponins.

AUC, area under the curve; NT-proBNP, N-terminal pro-B-type natriuretic peptide; ROC, receiver operating characteristic.

Table S2. Screening effects for ILR vs control according to ischemic stroke subtypes.

Outcome	ABC risk groups	Crude Event rate [95% CI] per 100 person-years		Hazard ratio [95% CI]	$p_{interaction}$
		Control	ILR		
**Ischemic stroke**	≤1%/year	0.77 [0.64-0.92]	0.63 [0.43-0.88]	0.81 [0.55-1.18]	0.5253
	>1%/year	1.45 [1.16-1.79]	0.95 [0.57-1.49]	0.66 [0.40-1.09]	
**Cardioembolic ischemic stroke or ESUS**	≤1%/year	0.29 [0.22-0.39]	0.24 [0.13-0.41]	0.81 [0.44-1.49]	0.9898
	>1%/year	0.68 [0.49-0.92]	0.55 [0.27-0.98]	0.81 [0.42-1.58]	
**Non-cardioembolic ischemic stroke**	≤1%/year	0.47 [0.37-0.58]	0.40 [0.25-0.61]	0.86 [0.54-1.39]	0.6130
	>1%/year	0.71 [0.52-0.96]	0.50 [0.24-0.91]	0.70 [0.35-1.39]	

Crude event rates are presented as event number per 100 person-years. Hazard ratios were estimated in a univariable cause-specific Cox regression model stratified for ABC-stroke risk group.

CI: confidence interval; ESUS, embolic stroke of undetermined source; ILR, implantable loop recorder.

Figure S1. Cumulative incidences of primary and secondary outcomes according to TnT subgroups

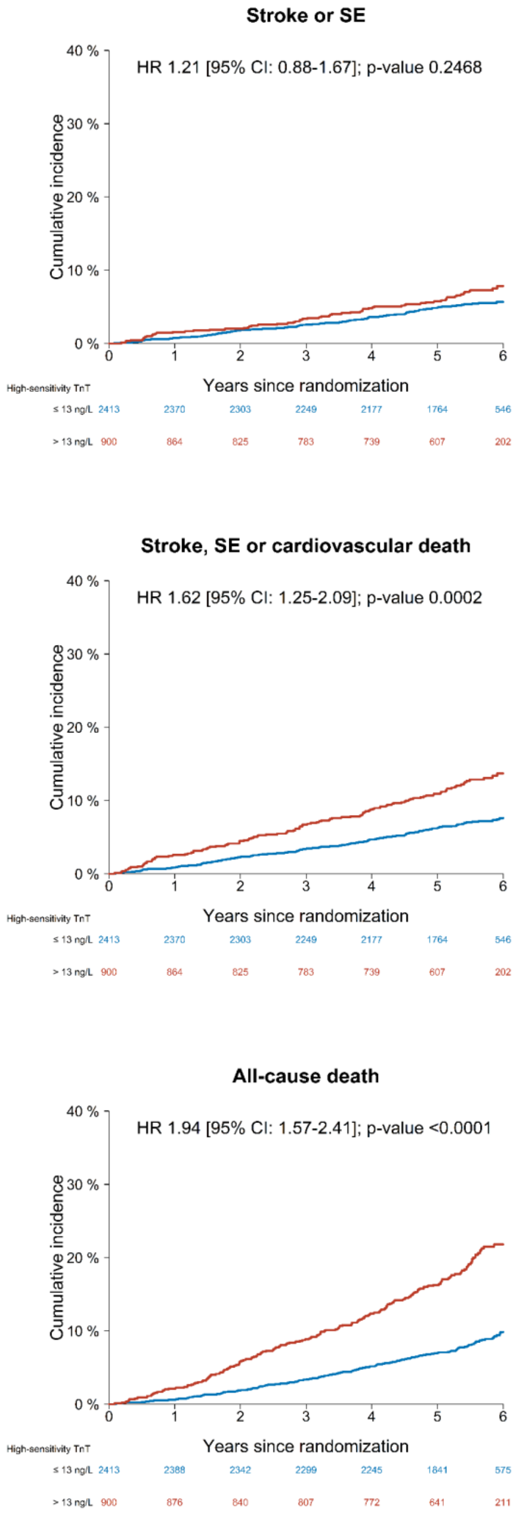

The figure shows the absolute risks of stroke/SE, stroke/SE/cardiovascular death, and all-cause death in the entire study cohort according to high-sensitivity TnT subgroups. Cumulative incidences were plotted using the Kaplan-Meier estimator for all-cause death and the Aalen-Johansen estimator for other outcomes with death as competing risk. HRs and p-values were determined in cause-specific Cox models adjusted for sex, age, body mass index,

weekly alcohol consumption, smoking pack years, hypertension, diabetes, prior stroke, heart failure, valvular heart disease, ischemic heart disease, and peripheral artery disease.

Cl, confidence interval; HR, hazard ratio; SE, systemic embolism; TnT, troponin T.

Figure S2. Cumulative incidences of primary and secondary outcomes according to TnI subgroups.

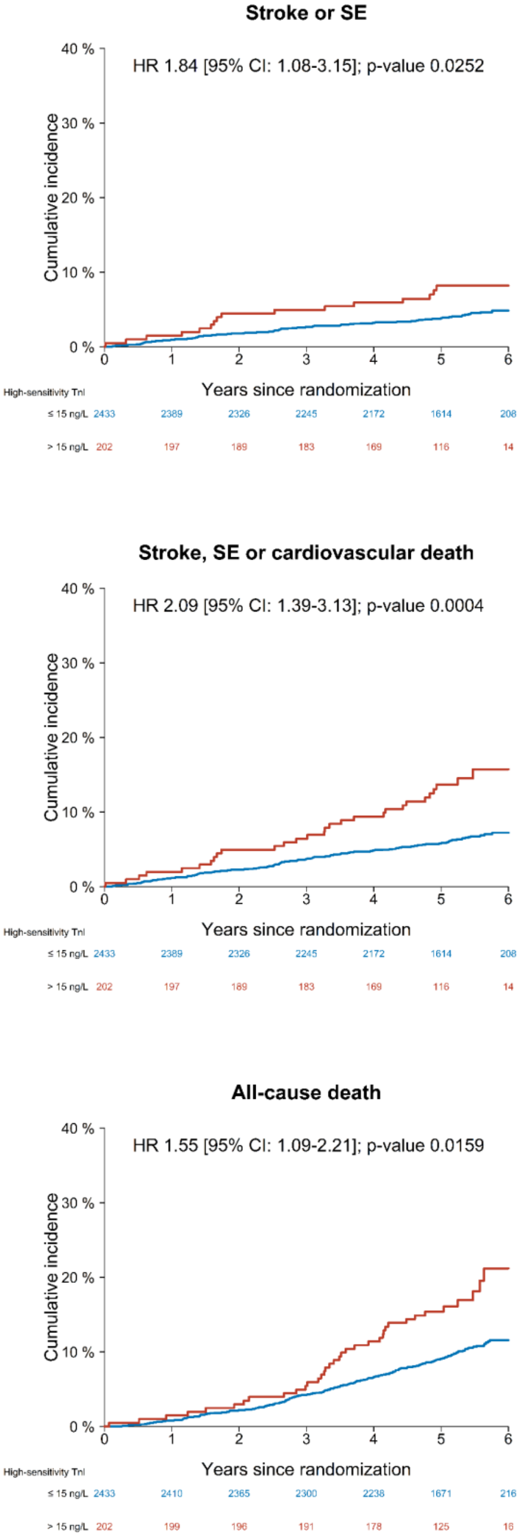

The figure shows the absolute risks of stroke/SE, stroke/SE/cardiovascular death, and all-cause death in the entire study cohort according to high-sensitivity TnI subgroups. Cumulative incidences were plotted using the Kaplan-Meier estimator for all-cause death and the Aalen-Johansen estimator for other outcomes with death as competing risk. HRs and p-values were determined in cause-specific Cox models adjusted for sex, age, body mass index,

weekly alcohol consumption, smoking pack years, hypertension, diabetes, prior stroke, heart failure, valvular heart disease, ischemic heart disease, and peripheral artery disease.

Cl, confidence interval; HR, hazard ratio; SE, systemic embolism; TnI, troponin I.

Figure S3. The associations of primary and secondary outcomes with continuous TnT

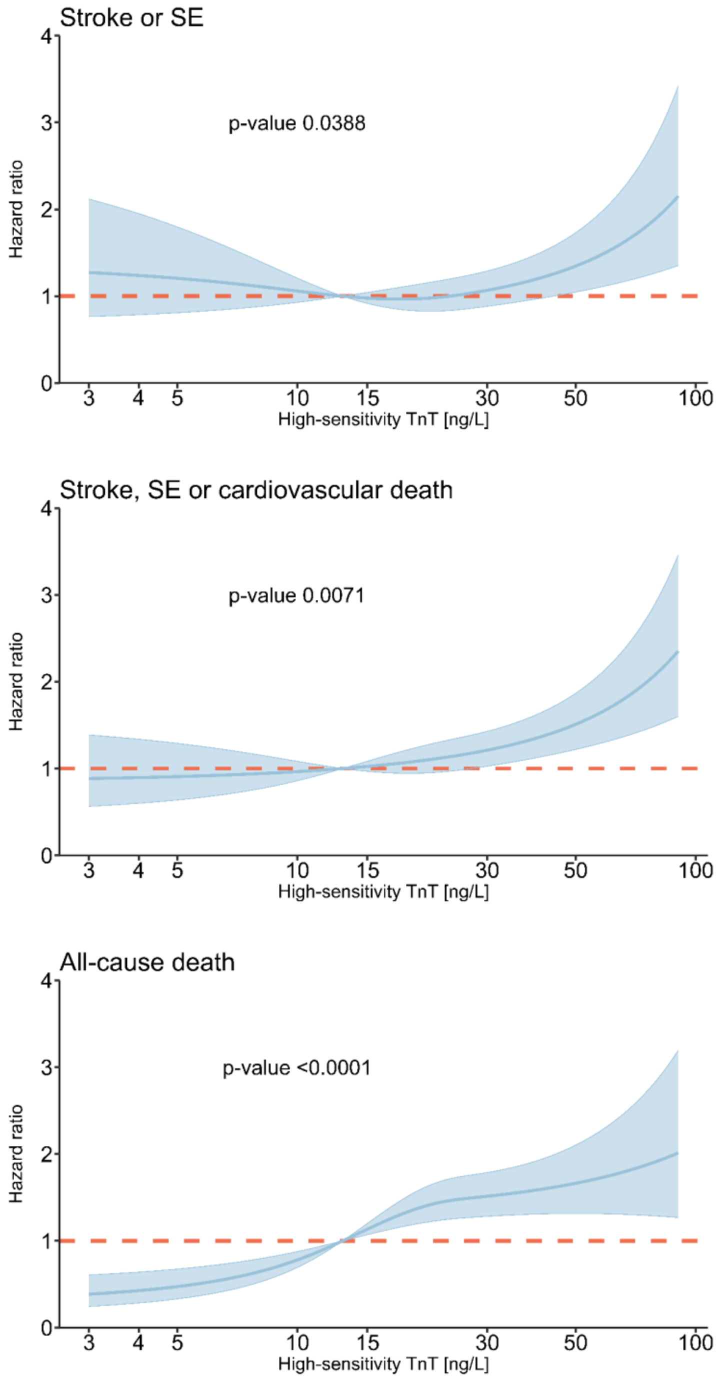

The figure shows the risks of stroke/SE, stroke/SE/cardiovascular death, and all-cause death in the entire study cohort according to high-sensitivity TnT as a continuous variable. Hazard ratios were determined with the median TnT value (13 ng/L) as reference, in cause-specific Cox models adjusted for sex, age, body mass index, weekly alcohol consumption, smoking pack years, hypertension, diabetes, prior stroke, heart failure, valvular heart disease, ischemic heart disease, and peripheral artery disease. The colored areas represent 95% confidence intervals.

SE, systemic embolism; TnT, troponin T.

Figure S4. The associations of primary and secondary outcomes with continuous TnI

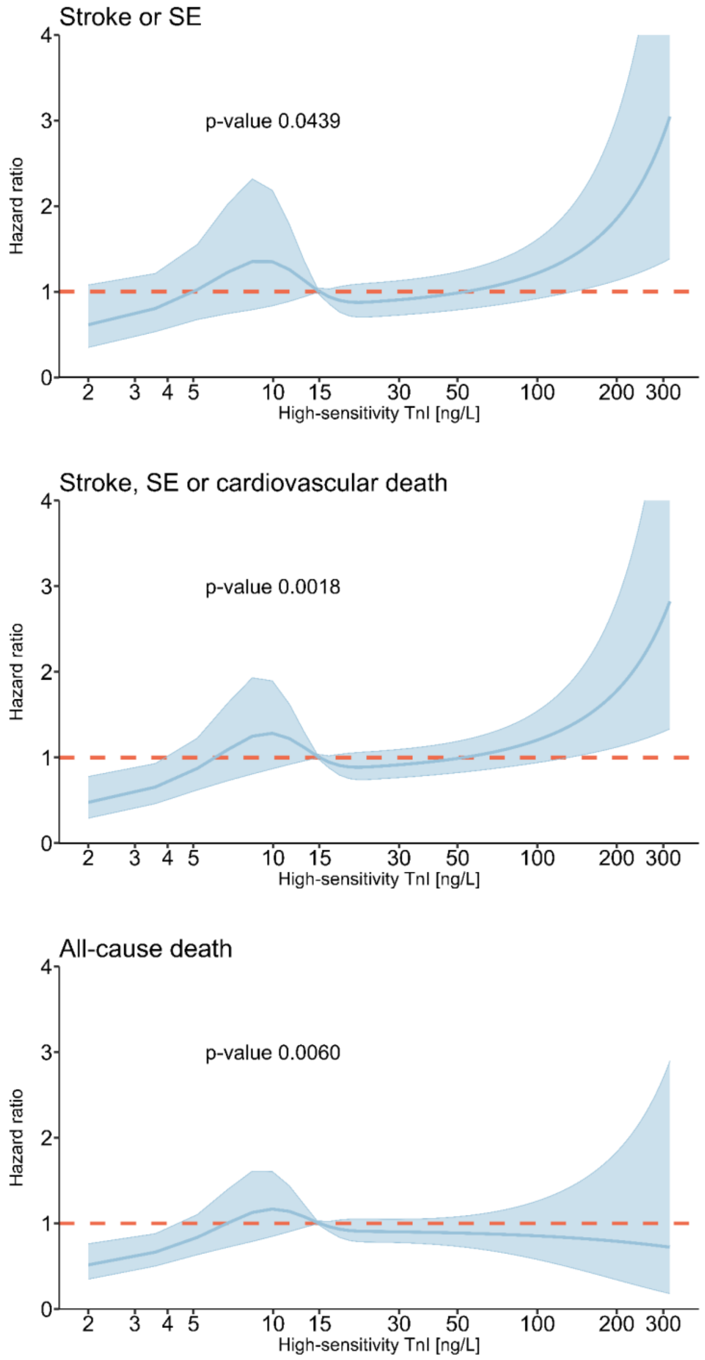

The figure shows the risks of stroke/SE, stroke/SE/cardiovascular death, and all-cause death in the entire study cohort according to high-sensitivity TnI as a continuous variable. Hazard ratios were determined with the median TnI value (15 ng/L) as reference, in cause-specific Cox models adjusted for sex, age, body mass index, weekly alcohol consumption, smoking pack years, hypertension, diabetes, prior stroke, heart failure, valvular heart disease, ischemic heart disease, and peripheral artery disease. The colored areas represent 95% confidence intervals.

SE, systemic embolism; TnI, troponin I.

Figure S5. The associations of primary and secondary outcomes with troponin percentile ranks.

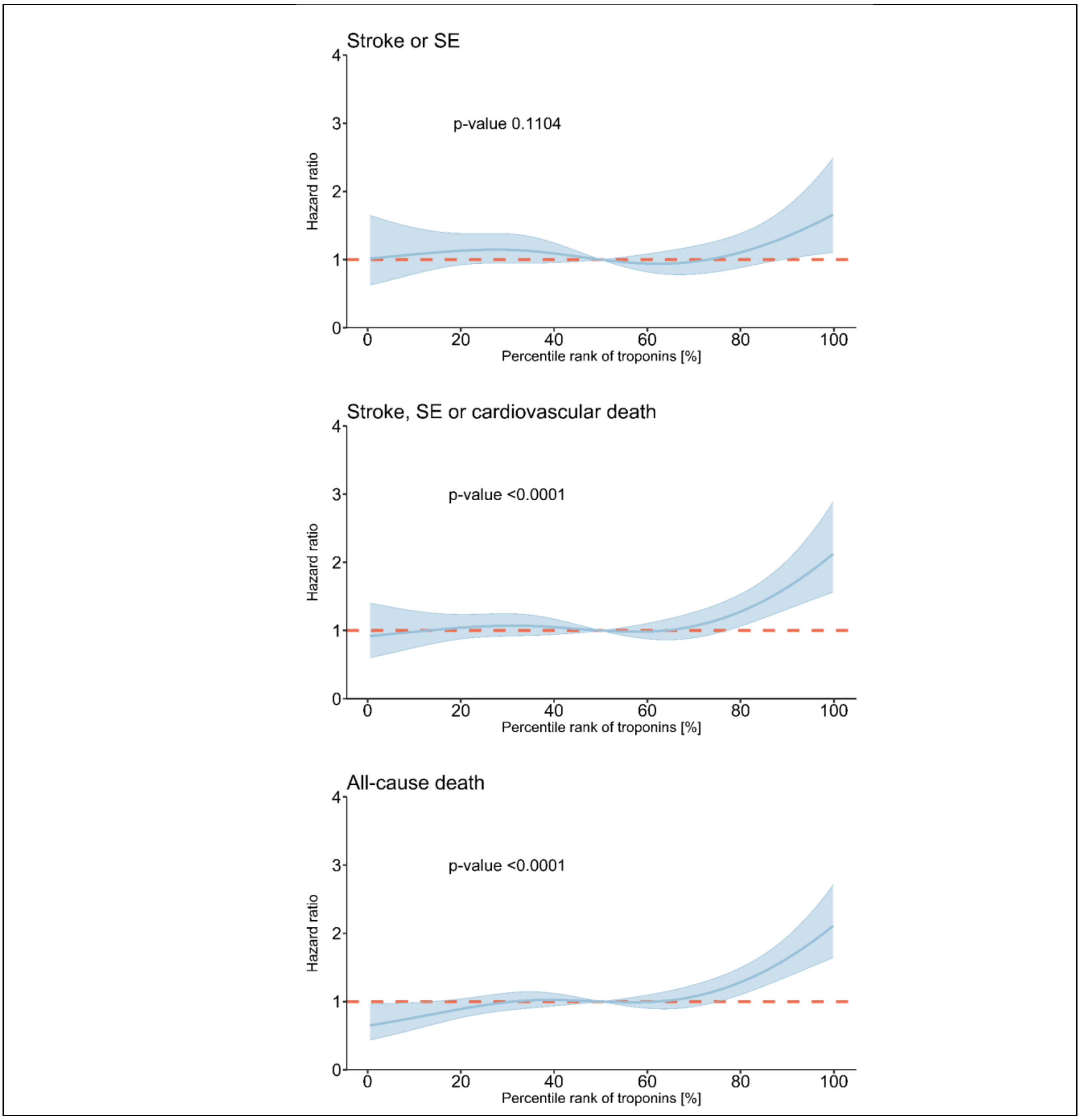

The figure shows the risks of stroke/SE, stroke/SE/cardiovascular death, and all-cause death in the entire study cohort according to high-sensitivity troponins. TnT and TnI were pooled by assigning a percentile rank to each participant, based on their TnT or TnI value in relation to the respective troponin distributions. Hazard ratios were determined with the 50th percentile rank of troponins as reference, in cause-specific Cox models adjusted for sex, age, body mass index, weekly alcohol consumption, smoking pack years, hypertension, diabetes, prior stroke, heart failure, valvular heart disease, ischemic heart disease, and peripheral artery disease. The colored areas represent 95% confidence intervals.

SE, systemic embolism; TnI, troponin I; TnT, troponin T.

Figure S6. The associations of ILR screening effects on primary and secondary outcomes with the estimated ABC-stroke risk at baseline.

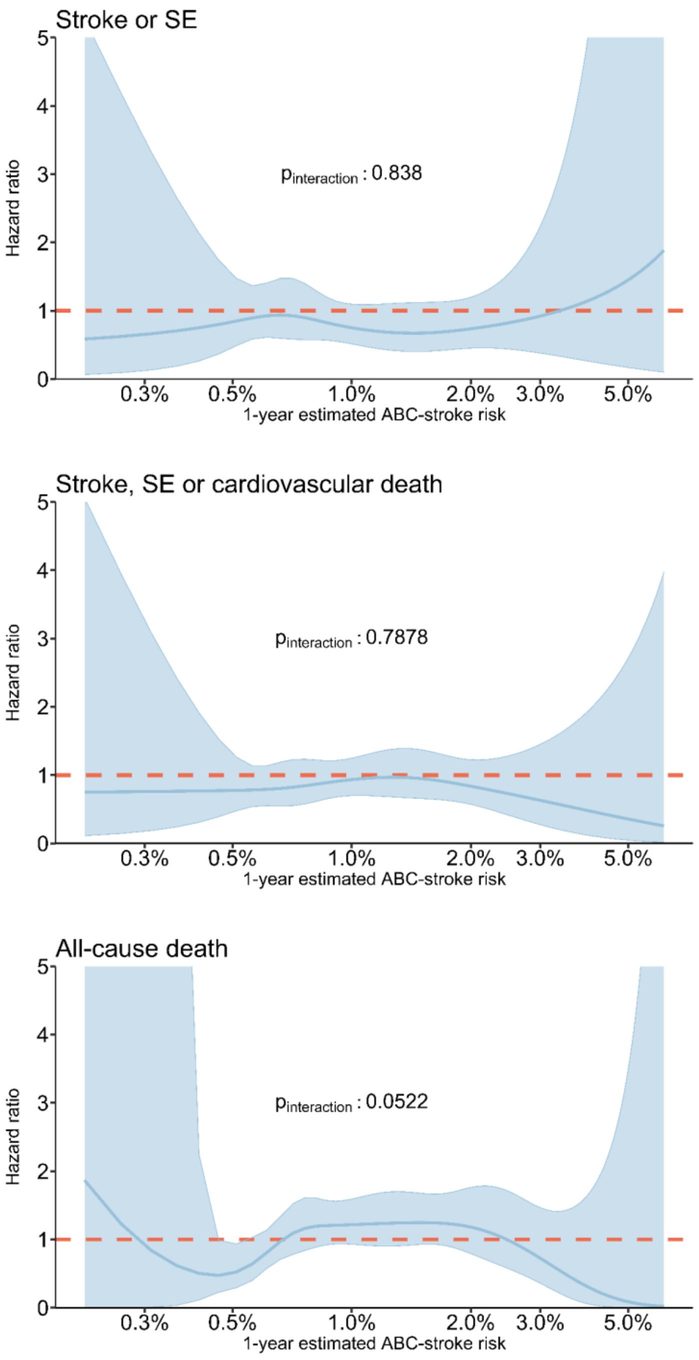

The figure shows the effects of ILR screening versus usual care on stroke/SE, stroke/SE/cardiovascular death, and all-cause death, according to the estimated ABC-stroke risk at baseline. Hazard ratios were estimated in cause-specific Cox models. The colored areas represent 95% confidence intervals.

ILR, implantable loop recorder; SE, systemic embolism.

Figure S7. The associations of ILR screening effects on primary and secondary outcomes with troponin percentile ranks.

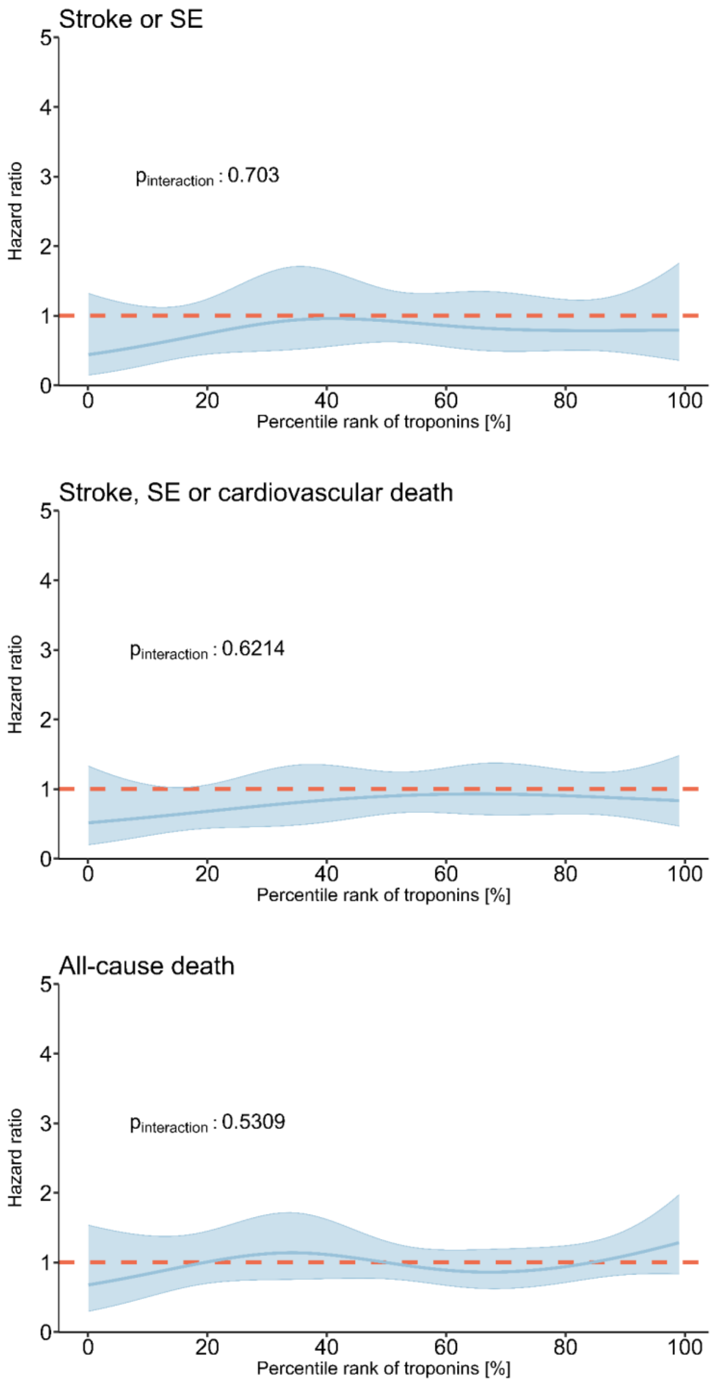

The figure shows the effects of ILR screening versus usual care on stroke/SE, stroke/SE/cardiovascular death, and all-cause death, according to high-sensitivity troponins. TnT and TnI were pooled by assigning a percentile rank to each participant, based on their TnT or TnI value in relation to the respective troponin distributions. Hazard ratios were estimated in cause-specific Cox models. The colored areas represent 95% confidence intervals.

ILR, implantable loop recorder; SE, systemic embolism; TnI, troponin I; TnT, troponin T.

Figure S8. Cumulative incidences of AF diagnosis according to TnT subgroups.

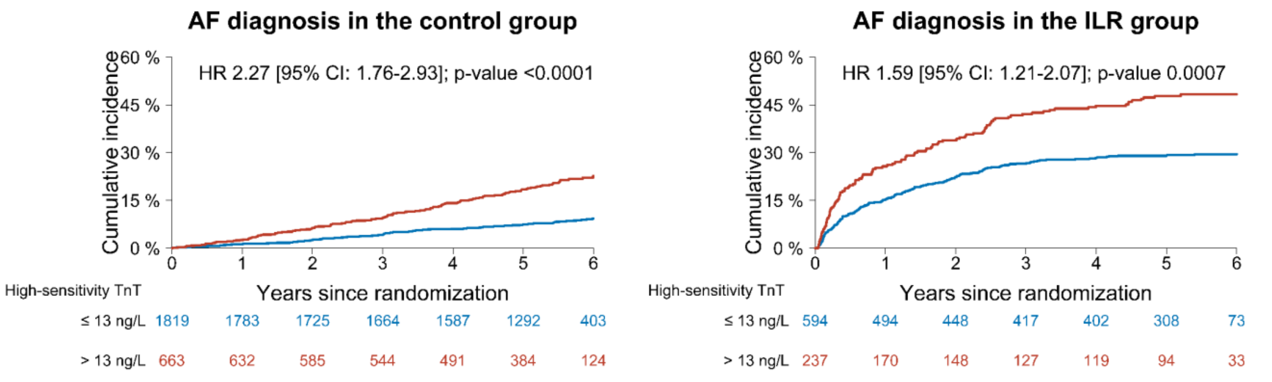

The figure shows the absolute risk of AF diagnosis in the control group and in the ILR group, according to high-sensitivity TnT subgroups. Cumulative incidences were plotted using the Aalen-Johansen estimator with death as competing risk. HRs and p-values were determined in cause-specific Cox models adjusted for sex, age, body mass index, weekly alcohol consumption, smoking pack years, hypertension, diabetes, prior stroke, heart failure, valvular heart disease, ischemic heart disease, and peripheral artery disease.

AF, atrial fibrillation; Cl, confidence interval; HR, hazard ratio; ILR, implantable loop recorder; TnT, troponin T.

Figure S9. Cumulative incidences of AF diagnosis according to TnI subgroups.

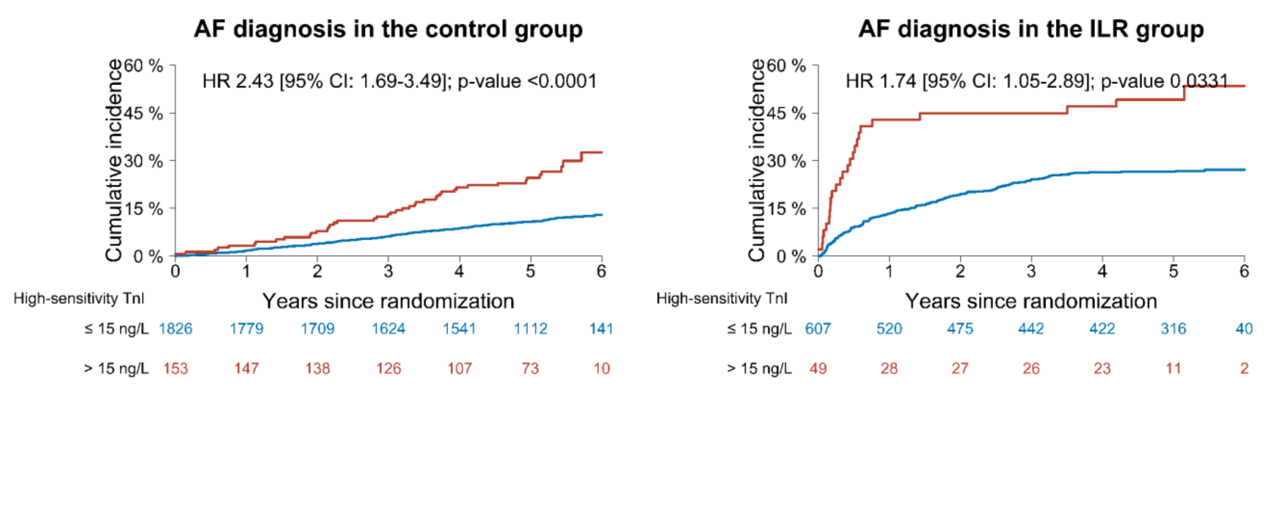

The figure shows the absolute risk of AF diagnosis in the control group and in the ILR group, according to high-sensitivity TnI subgroups. Cumulative incidences were plotted using the Aalen-Johansen estimator with death as competing risk. HRs and p-values were determined in cause-specific Cox models adjusted for sex, age, body mass index, weekly alcohol consumption, smoking pack years, hypertension, diabetes, prior stroke, heart failure, valvular heart disease, ischemic heart disease, and peripheral artery disease.

AF, atrial fibrillation; Cl, confidence interval; HR, hazard ratio; ILR, implantable loop recorder; TnI, troponin I.
